# Seven N-terminal Residues of a Thermophilic Xylanase Are Sufficient to Confer Hyperthermostability on Its Mesophilic Counterpart

**DOI:** 10.1371/journal.pone.0087632

**Published:** 2014-01-30

**Authors:** Shan Zhang, Yongzhi He, Haiying Yu, Zhiyang Dong

**Affiliations:** State Key Laboratory of Microbial Resources, Institute of Microbiology, Chinese Academy of Sciences, Beijing, P. R. China; Jacobs University Bremen, Germany

## Abstract

Xylanases, and especially thermostable xylanases, are increasingly of interest for the deconstruction of lignocellulosic biomass. In this paper, the termini of a pair of xylanases, mesophilic SoxB and thermophilic TfxA, were studied. Two regions in the N-terminus of TfxA were discovered to be potentially important for the thermostability. By focusing on Region 4, it was demonstrated that only two mutations, N32G and S33P cooperated to improve the thermostability of mesophilic SoxB. By introducing two potential regions into SoxB in combination, the most thermostable mutant, M2-N32G-S33P, was obtained. The M2-N32G-S33P had a melting temperature (Tm) that was 25.6°C higher than the Tm of SoxB. Moreover, M2-N32G-S33P was even three-fold more stable than TfxA and had a Tm value that was 9°C higher than the Tm of TfxA. Thus, for the first time, the mesophilic SoxB “pupil” outperformed its thermophilic TfxA “master” and acquired hyperthermostability simply by introducing seven thermostabilizing residues from the extreme N-terminus of TfxA. This work suggested that mutations in the extreme N-terminus were sufficient for the mesophilic xylanase SoxB to acquire hyperthermostability.

## Introduction

Xylanases are increasingly recognized to be important for the deconstruction of lignocellulosic biomass [1], [2]. Xylanases not only catalyze the hydrolysis of hemicelluloses but also render cellulose more accessible to enzymatic hydrolysis [3]–[6]. Thermostable xylanases possess obvious advantages over their counterparts because of the reduced associated costs and their ability to be manipulated for the deconstruction of lignocellulosic biomass [7], [8]. It is not surprising, therefore, that widespread research endeavors have focused on developing thermostable xylanases by rational design or directed evolution.

The rational design strategy of replacing the N-terminus of mesophilic xylanases with the first 31 residues of the thermophilic xylanase TfxA from *Thermomonospora fusca* has successfully produced several thermostable hybrid xylanases, such as Stx15 [9], StxAB [10], Btx [11] and ATx [12]. These hybrid xylanases exhibited higher thermostabilities than their corresponding mesophilic parents. However, compared with TfxA, all of the hybrid xylanases displayed significantly inferior thermostabilities. Recently, another hybrid xylanase AEx11A was designed by substituting N-terminus of AoXyn11A with the corresponding region of a hyperthermostable xylanase, *Ev*Xyn11^TS^ [13]. Likewise, the thermostability of the hybrid xylanase was higher than that of mesophilic parent AoXyn11A, but still significantly lower than that of thermophilic parent *Ev*Xyn11^TS^ [13], [14]. That is to say, all these hybrid xylanases containing the N-terminus of thermophilic xylanases displayed improved thermostabilities, but could not outperform their thermophilic parents to acquire hyperthermostabilities. Thus, a both industrially and biologically intriguing and important question arose: Are mutations in the extreme N-terminus sufficient for conferring hyperthermostability on mesophilic xylanases?

Directed evolution has been successfully applied to confer thermostability (or even hyperthermostability) on mesophilic xylanases. Using this technique, Ruller et al. [15] improved the melting temperature (Tm) of xylanase A from *Bacillus subtilis* from 59°C to 76.5°C. This mutant xylanase contained four mutations (*Q7H*, *G13R*, *S22P* and *S179C*), of which three were in the extreme N-terminus. Palackal et al. [16] also employed directed evolution and successfully obtained a thermostable variant (9X) with a high Tm (95.6°C). The 9X enzyme harbored nine mutations, of which four (*D8F*, *Q11H*, *N12L* and *G17I*) were in the extreme N-terminus. Likewise, Miyazaki et al. [17] created one thermostable variant, Xyl^st^, by directed evolution. This mutant contained three amino acid substitutions, of which two were in the extreme N-terminus. In addition, Dumon et al. [14] successfully engineered a hyperthermostable GH11 xylanase, *Ev*Xyn11^TS^. The Tm of *Ev*Xyn11^TS^ was approximately 25°C higher than the Tm of the parent xylanase. It contained seven mutations, of which six (*S9P*, *T13F*, *N14H*, *Y18F*, *Q34L* and *S35E*) were in the extreme N-terminus. During the course of directed evolution, the importance of thermostabilizing mutations in the extreme N-terminus was highlighted. However, concomitant thermostabilizing mutations outside of the extreme N-terminus obscured the significance of the extreme N-terminal mutations in conferring thermostability. Thus, the same question remained: Would it be possible for mutations in the extreme N-terminus to confer hyperthermostability on mesophilic xylanase?

Moreover, it should be noted that several attempts have been made to obtain hyperthermostable xylanases by engineering thermophilic xylanases [18]–[22]. Using thermophilic xylanases, it is not difficult to obtain hyperthermostable mutants. However, for mesophilic xylanases, achieving hyperthermostability is a much bigger challenge.

In this work, by focusing on the extreme N-termini, a convenient and straightforward approach to obtain hyperthermostable xylanases was attained. Additionally, mutations in only the extreme N-terminus were confirmed to be sufficient to confer thermostability (or even hyperthermostability) on mesophilic SoxB.

## Materials and Methods

### Strains and Growth Conditions

*Escherichia coli* TOP10 and *E. coli* BL21 (DE3) were used for the cloning and heterologous expression, respectively, of xylanase genes. The *E. coli* was cultured in Luria-Bertani (LB) broth at 37°C.

*Streptomyces olivaceoviridis* (ATCC 23630) and *T. fusca* (ATCC 27730) were grown in GYM S*treptomyces* medium (0.4% glucose, 0.4% yeast extract, 1% malt extract and 0.2% CaCO_3_) at 28°C and TYG medium (0.3% tryptone, 0.3% yeast extract, 0.3% glucose and 0.1% K_2_HPO_4_) at 50°C, respectively.

### DNA Manipulation and Plasmid Constructs

The genomic DNA of *S. olivaceoviridis* and *T. fusca* were used as polymerase chain reaction (PCR) templates to amplify the *SoxB* (GenBank accession mumber KF927165) and *TfxA* (GenBank accession mumber KF927166) genes. The region mutants of SoxB were constructed by overlap extension PCR [23]. The primers used in this study are listed in Table S1. All of the genes from the wildtype and region-mutant xylanases were digested with *Nde*I and *Eco*RI (TaKaRa) and ligated into the pET-28a (+) vector (Novagen). Each mutation was confirmed by sequencing of the entire xylanase gene.

### Expression and Purification of Recombinant Xylanases

The recombinant plasmids containing the wildtype and mutant xylanases were transformed into *E. coli* BL21 (DE3). Overnight cultures of *E. coli* BL21 (DE3) harboring the recombinant plasmids were diluted 1/100 in fresh LB containing 50 µg/ml kanamycin. The cultures were incubated at 37°C and shaken at 200 rpm until an absorbance between 0.6 and 0.8 at 600 nm was reached. The cultures were then induced with 0.5 mM isopropyl-β-D-thiogalactopyranoside (IPTG). Following 6 h of induction at 30°C, the cells were harvested by centrifugation at 6,000 rpm for 20 min at 4°C. After the pellet was resuspended in binding buffer (50 mM sodium phosphate [pH 7.4] and 500 mM sodium chloride), the cells were lysed ultrasonically. The insoluble material was removed by centrifugation at 12,000 rpm for 20 min at 4°C, and the supernatant was then applied to a Ni-NTA-agarose affinity column and purified by fast protein liquid chromatography (FPLC). The protein was washed with binding buffer containing 100 mM imidazole and eluted with 400 mM imidazole in binding buffer. The purified xylanase was pooled and dialyzed to remove the imidazole. For both the wildtype and all of the mutant xylanases, a single-step cation-exchange purification protocol yielded pure protein, as evaluated by Coomassie-stained sodium dodecyl sulphate-polyacrylamide gel electrophoresis (SDS-PAGE) [24]. The protein concentration was determined using the Pierce bicinchoninic acid (BCA) protein reagent (Thermo Scientific) and including bovine serum albumin (BSA) as the protein standard.

### Enzyme Activity Assay

Xylanase activity was determined using the dinitrosalicylic acid (DNS) method, with a few modifications [25]. The assay was performed in 50 mM sodium citrate buffer (pH 6.0), using birch wood xylan (Sigma-Aldrich) as the substrate. Initially, 40 µl of diluted enzyme was mixed with 360 µl of 10% birch wood xylan and incubated for 10 min at 50°C. Next, 600 µl of DNS reagent was added, and the solution was heated to 99°C for 15 min. The enzyme activity was determined from the increase in absorbance at 540 nm measured by a Sunrise Microplate Reader (TECAN). One international unit of enzyme activity was defined as the amount of enzyme necessary to liberate 1 µmol of reducing sugars per minute under the assay conditions. The optimum temperature for the enzyme activity was determined by a standard assay at various temperatures in 50 mM sodium citrate buffer (pH 6.0). The results were expressed as the activity relative to the value obtained from the optimum temperature assay.

### Thermal Inactivation Assay

Thermal inactivation assays were performed in 50 mM sodium citrate buffer (pH 6.0) at 70°C or 80°C in the absence of the substrate. At specific intervals, 40 µl aliquots were removed and cooled on ice. The residual activity was measured as described above for the enzyme activity measurement. The half-life curves were fitted to equation *y* = A*e^−k^ ^t^ and the half-life was then calculated from the thermal inactivation curve, as previously described [10].

### Differential Scanning Calorimetry (DSC) Experiments

DSC experiments were performed using a Nano DSC (TA Instruments) with sample concentrations of 1 mg/ml and a scan rate of 1°C/min, as previously described [10], [26]. The instrumental baseline, with both cells filled with buffer, was also routinely recorded before experiments.

### Sequence Alignment and Homology Modelling

Sequence alignment was performed using the DNAman software package (Lynnon Biosoft). The homology model of SoxB and its mutants was developed using the software package modeller [27], [28]. Three crystal structures (PDB codes: 1HIX, 1M4W and 3ZSE) were chosen as the templates. The structure visualisation was performed using Pymol [29] software.

## Results and Discussion

### Identification of Two Potential Regions in the Extreme N-terminus

**Figure 1:**
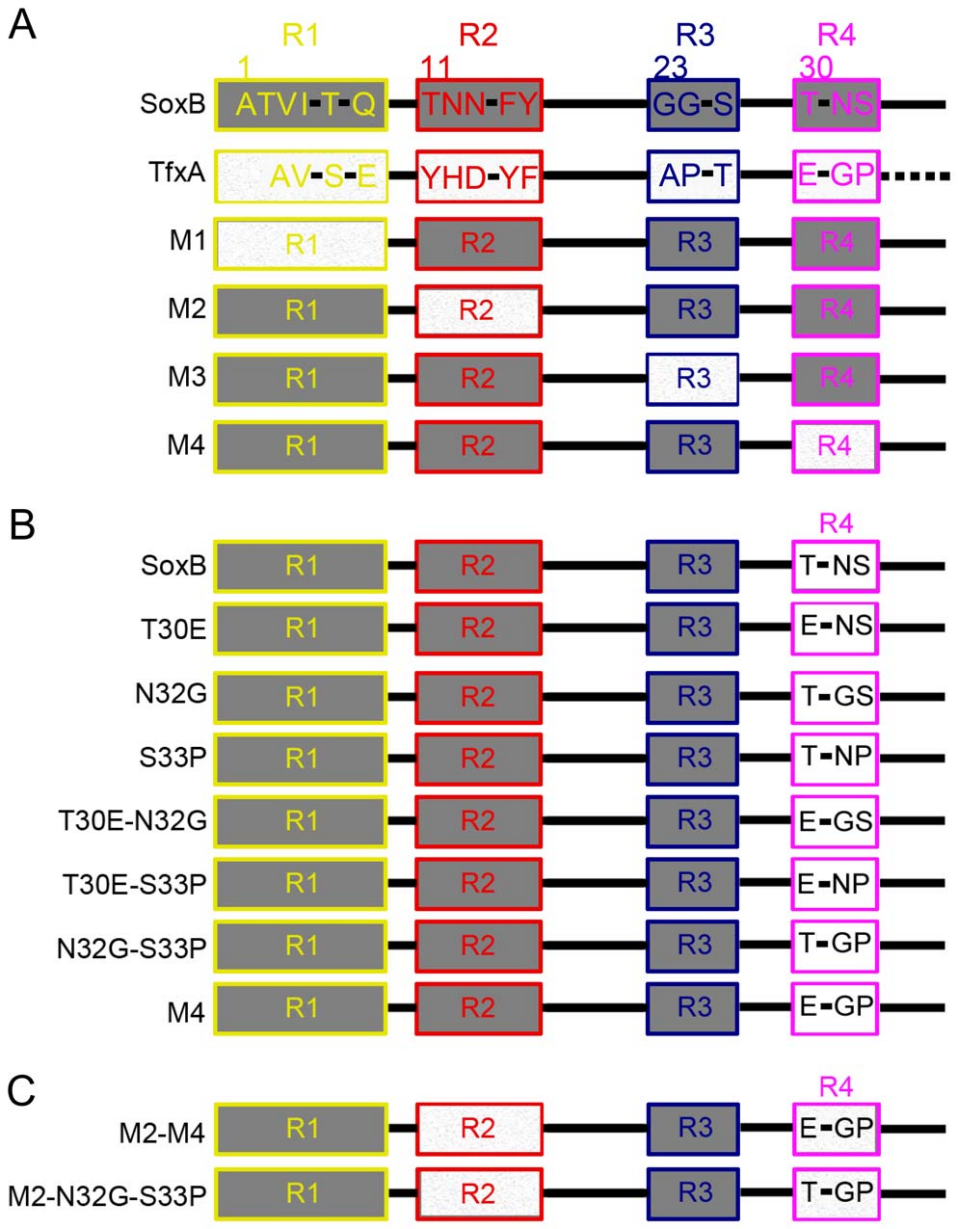
Schematic representation of widetype xylanases and mutants. (A) Region mutants were produced according to the divergent regions between the N-termini of SoxB and TfxA. (B) Focusing on R4, three mutants (T30E, N32G and S33P), each containing a single mutation and three double mutants (T30E-N32G, T30E-S33P and N32G-S33P) were created. (C) Two double region mutants were produced by introducing two potential regions into SoxB.

**Figure 2:**
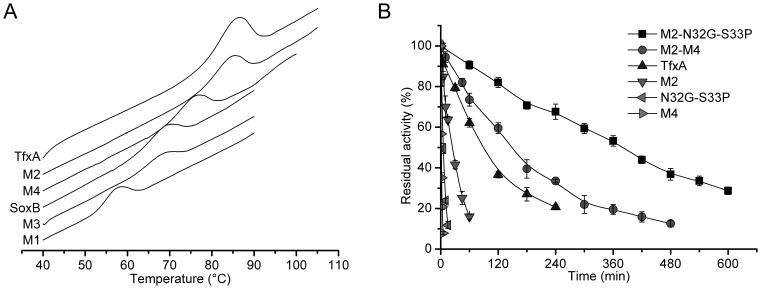
Comparison of the thermostabilities of SoxB, TfxA and region mutants. (A) The DSC profiles. (B) The thermal inactivation profiles at 80°C.

The amino acid sequence of the mesophilic xylanase SoxB shares high-level identity (74%) with the sequence of the catalytic domain of the thermophilic homolog TfxA. However, this pair of xylanases exhibits only approximately 50% identity for the first 33 N-terminal residues (numbered by the SoxB sequence) (Fig. 1A and Fig. S1). As we demonstrated in our previous study [10], based on sequence alignment and structural analysis, the divergent positions of the 33 N-terminal residues were clustered in four regions (R1, R2, R3 and R4) (Fig. 1A). Accordingly, four region mutants of SoxB (M1, M2, M3 and M4) were produced by substituting the individual regional residues of SoxB with the corresponding regional residues in TfxA (Fig. 1A). Here, the thermostabilities of these four region mutants were compared with not only the thermostability of SoxB but also the thermostability of TfxA. Both DSC and thermal inactivation experiments demonstrated that the rank order of the thermostabilities was as follows: TfxA>M2> M4> SoxB>M3> M1 (Fig. 2). The importance of Region 2 for thermostability has been discussed in our previous study [10]. More specifically, we demonstrated that all five of the divergent residues in R2 of TfxA were essential and played a synergistic role in conferring thermostability. Here, the interest was focused on R4. The Tm of the region mutant M4 was 6.7°C higher than the Tm of SoxB (Fig. 2A). Moreover, at 70°C, the M4 displayed a nine-fold higher thermostability than SoxB (Table 1). Thus, the residues in R4 of TfxA were also important for conferring thermostability. However, it should be noticed that all the four region mutants displayed significantly lower thermostabilities than thermophilic TfxA. Even the M2 and M4 mutants exhibited four-fold and 70-fold lower thermostability at 80°C than thermophilic TfxA, respectively (Fig. 2B).

**Table 1:** Systematic analysis and comparison of the thermostabilities of mutants in R4.

Xylanases	Mutations	t_1/2_ at70°C (min)	Tm (°C)	ΔTm
SoxB	–	3.6±0.1	69.1	–
M4	*T30E, N32G* and *S33P*	33±1.6	75.8	6.7
T30E	*T30E*	1.8±0.1	65.8	–3.3
N32G	*N32G*	17±0.2	74.9	5.8
S33P	*S33P*	2.2±0.1	67.0	–2.1
T30E-N32G	*T30E* and *N32G*	10±0.1	72.2	3.1
N32G-S33P	*N32G* and *S33P*	87±4.5	78.2	9.1
T30E-S33P	*T30E* and *S33P*	1.4±0.1	64.3	–4.8

In summary, R2 and R4 of TfxA were confirmed to be two potential regions for conferring thermostability. Two region mutants, M2 and M4 mutants displayed higher thermostability than their mesophilic parent SoxB. However, compared with their thermophilic parent TfxA, all the region mutants displayed inferior thermostability.

### The Effect of Each Mutation in Region 4 on Thermostability

**Figure 3:**
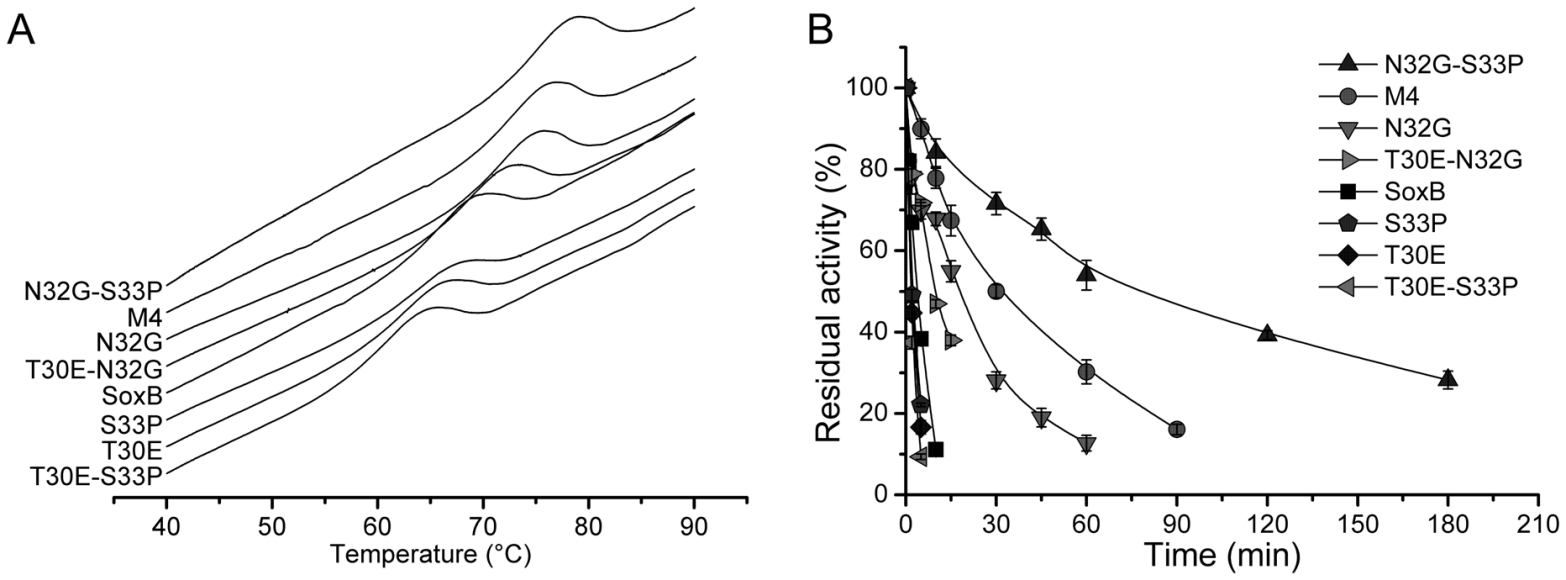
Analysis of the effect of mutations in R4 on thermostability. (A) DSC profiles. (B) The thermal inactivation profiles at 70°C for the mutants.

As shown in Fig. 1A, three residues that diverged between SoxB and TfxA were found in R4. Therefore, three mutants of SoxB (T30E, N32G and S33P), each containing a single mutation, were generated (Fig. 1B). To our surprise, two of the three mutants, T30E and S33P, decreased the thermostability of SoxB rather than increasing this parameter. Only the N32G mutant displayed a higher thermostability than SoxB (Fig. 3A and Fig. 3B). Thus, the *N32G* mutation was of vital importance in conferring thermostability. However, compared with the M4 mutant, the N32G mutant still exhibited a lower thermostability (Fig. 3A and Fig. 3B), which suggested a potential synergistic effect between the three mutations (*N32G*, *T30E* and *S33P*).

### *N32G* and *S33P* in Region 4 Displayed Synergistic Effects

To discern the potential synergistic effect between the *N32G*, *T30E* and *S33P* mutations, three double mutants (T30E-N32G, T30E-S33P and N32G-S33P) were produced and their thermostabilities were analyzed (Fig. 1B).

First, the two double mutants harboring the *N32G* mutation, T30E-N32G and N32G-S33P, displayed a higher thermostability than the T30E and S33P mutants, respectively (Fig. 3A and Fig. 3B). This finding once again strongly indicated the importance of the *N32G* mutation in conferring thermostability. More importantly, the N32G-S33P mutant exhibited an approximately 40-fold and five-fold higher thermostability than S33P and N32G, respectively. And the Tm of the N32G-S33P mutant was also 11.2°C and 3.3°C higher than the Tm values of S33P and N32G, respectively (Table 1). Furthermore, to our surprise, the N32G-S33P mutant displayed even higher thermostability than M4 (Fig. 3A, Fig. 3B and Table 1). All these results clearly revealed a synergistic effect between N32G and S33P.

In contrast, the two double mutants harboring the *T30E* mutation, T30E-N32G and T30E-S33P, exhibited a lower thermostability than N32G and S33P, respectively (Fig. 3A and Fig. 3B and Table 1). This result agreed well with the negative effect of single mutation *T30E* on thermostability and demonstrated that the *T30E* mutation was unfavourable for the thermostability of SoxB.

As for the two double mutants containing the *S33P* mutation, the N32G-S33P mutant displayed a higher thermostability than the N32G mutant, whereas T30E-S33P exhibited a lower thermostability than the T30E mutant (Fig. 3A, Fig. 3B and Table 1). This finding appeared to be paradoxical at first but provided important information: the *S33P* was a thermostabilizing mutation only when combined with the *N32G* mutation, which further demonstrated the synergistic effect between *N32G* and *S33P*. Dumon [14] employed gene site saturation mutagenesis™ (GSSM) to discover all of the possible individual thermostabilizing mutations in *Ev*Xyn11^TS^. Our result explains why the corresponding mutation of *S33P* was not detected as a thermostabilizing mutation in *Ev*Xyn11^TS^, although the corresponding mutation of *N32G* (N30V) was discovered to be thermostabilizing.

**Figure 4:**
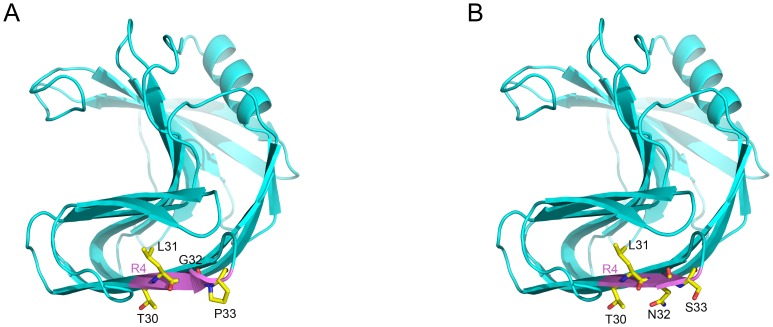
Homology modelling and structure comparison of the N32G-S33P mutant (A) and wild type SoxB (B). The Region 4 was in purple, and the residues in R4 were in yellow.

To understand the structural basis of the synergistic effect between *N32G* and *S33P*, a structural model for N32G-S33P mutant was generated and analyzed (Fig. 4). The pyrrolidine ring restricts proline to fewer conformations than are available to the other amino acids [30], [31]. Thus, proline is believed to strengthen the rigidity of proteins [32]–[34]. When the single *S33P* mutation was introduced into the sequence of SoxB, the Asn32 residue, which is immediately adjacent to Pro33, may have restrained the local configuration of Asn32 and Pro33. Thus, the single *S33P* mutation may have impaired the local configuration of SoxB and hence decreased the thermostability of SoxB. Unlike Asn32, glycine lacks a β-carbon and has more backbone conformational flexibility. Therefore, in N32G-S33P mutant, the *N32G* mutation would help Pro33 to form the desirable configuration, which would strengthen the rigidity of R4 of SoxB and improve the thermostability (Fig. 4A).

### Two Potential Regions in Combination Conferred Hyperthermostability on SoxB

**Figure 5:**
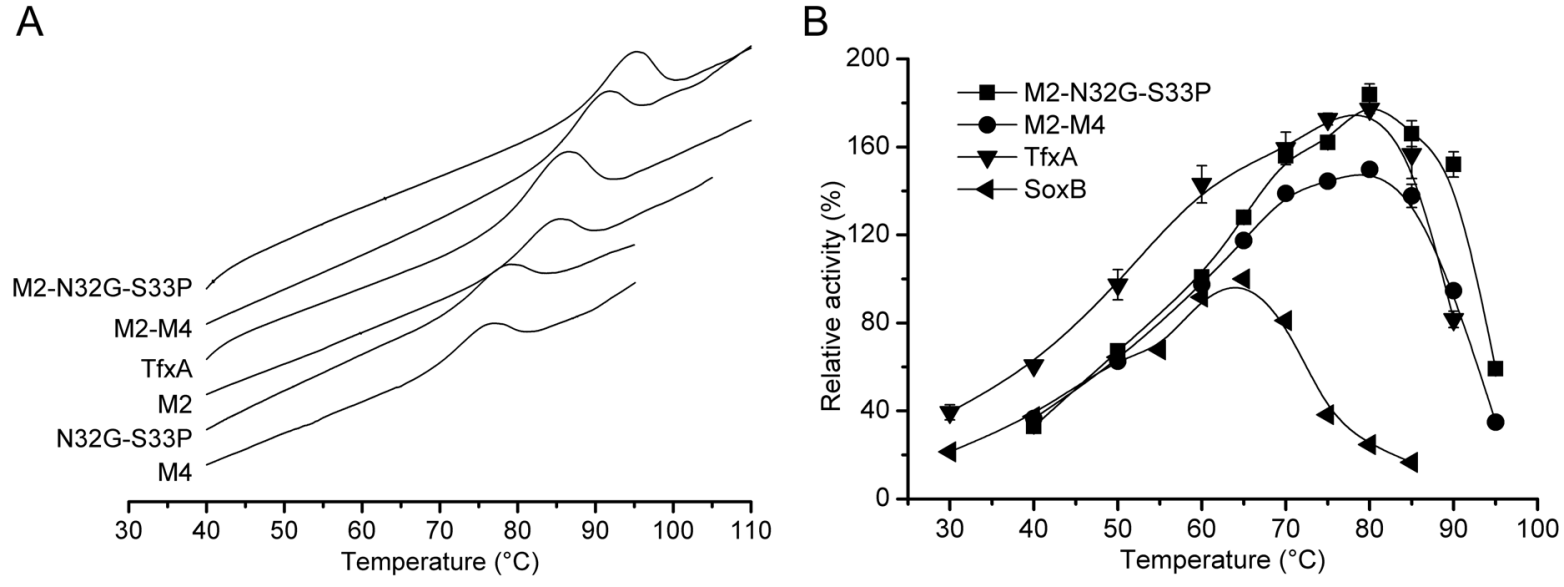
Comparison of thermostabilities of double region mutants, M2-M4 and M2-N32G-S33P. (A) DSC profiles. (B) The optimum temperature profiles.

To test whether the thermostability of SoxB was further enhanced by introducing mutations in both R2 and R4 simultaneously, two double region mutants, M2–M4 and M2–N32G-S33P, were produced (Fig. 1C). The M2–M4 mutant contained five mutations (*T11Y*, *N12H*, *N13D*, *F15Y* and *Y16F*) in Region 2 and three mutations (*T30E*, *N32G* and *S33P*) in Region 4, and M2-N32G-S33P harbored five mutations (*T11Y*, *N12H*, *N13D*, *F15Y* and *Y16F*) in Region 2 and the two mutations (*N32G* and *S33P*) in Region 4. Both the M2-M4 and M2-N32G-S33P mutants had a significantly higher thermostability than M2, M4 and N332G-S33P, suggesting that the thermostability of SoxB was significantly further enhanced. Due to the negative effect of the *T30E* mutation on thermostability, it was not surprising that the M2-M4 mutant displayed a lower thermostability than M2-N32G-S33P (Fig. 2B, Fig. 5A and Table 2).

**Table 2:** Comparsion of the thermostabilities of double region mutants with wildtype parents, SoxB and TfxA.

Xylanases	t_1/2_ at 80°C (min)	Tm (°C)	ΔTm	Optimum temperature (OT) (°C)	Specific activity at OT(IU/mg)
SoxB	–a	69.1	–	65	801
TfxA	94±6	85.7	16.6	80	1419
M2-M4	151±6	90.9	21.8	80	1200
M2-N32G-S33P	354±21	94.7	25.6	80	1472

a The xylanase lost all of its enzyme activity within only 1 min so that the t_1/2_ could not be determined.

The M2-N32G-S33P mutant exhibited the highest thermostability among all of the mutants derived from SoxB. The Tm of M2-N32G-S33P was 94.7°C, which was 25.6°C higher than the Tm of SoxB. Furthermore, the M2-N32G-S33P mutant had an increased half-life at 80°C, which indicated 71-fold and 15-fold more stability than the half-life of N32G-S33P and M2, respectively (Fig. 2B, Fig. 5A and Table 2). In contrast, the SoxB lost its activity at 80°C so rapidly that the half-life could not be determined. Furthermore, the optimum temperature of M2-N32G-S33P was 80°C, which was 15°C higher than the optimum temperature of wildtype SoxB, and the specific activity of M2-N32G-S33P at this temperature (80°C) was 1.8-fold higher than the activity of wildtype SoxB at its optimum temperature (65°C) (Fig. 5B and Table 2). Thus, the seven mutations in the extreme N-terminus, which is located far from the catalytic centre of SoxB, did not compromise, and in fact improved, enzyme activity.

Because the seven or eight residues substituted in M2-N32G-S33P and M2-M4 were totally derived from TfxA, the thermostabilities of M2-N32G-S33P and M2-M4 were compared with the thermostability of TfxA. Intriguingly, both the M2-M4 and the M2-N32G-S33P mutants yielded an even higher thermostability than TfxA. In particular, M2-N32G-S33P was three-fold more stable than TfxA, and the Tm of M2-N32G-S33P was 9°C higher than the Tm of TfxA (Fig. 2B, Fig. 5A and Table 2). Thus, the mesophilic xylanase “pupil” outperformed its thermophilic “master” to acquire hyperthermostability simply by having seven mutations introduced into the extreme N-terminus. The specific activities of TfxA and M2-N32G-S33P were measured at 80°C. The M2-N32G-S33P had a quite comparable specific activity with that of TfxA (Fig. 5B and Table 2). The temperatures used for hydrolysis of heterologous xylans by xylanases may vary in different fields including food, animal feed, biofuel or paper production. In the field of biofuel, currently most plant cell wall polysaccharides-degrading enzymes including xylanases are derived from the mesophilic filamentous fungus *Trichoderma reesei*. Normally, these enzymes are used at around 50°C. It is expected that higher temperatures will be more beneficial at least for decreasing the viscosity of culture fluids, minimizing the possibility of microbial contamination, and facilitating the distillation of bioethanol. Therefore, there are tremendous efforts in discovering novel glycoside hydrolases [35] or engineering existing ones for better tolerance of higher temperatures [36]. This is also one of the purposes of current study. However, an industrial standard has not been settled yet for the reaction temperatures of all these thermophilic enzymes published previously. Here, the specific activities of TfxA and M2-N32G-S33P were also compared at a decreased temperature of 70°C, which is 10°C lower than the optimal temperatures of the M2-N32G-S33P and the TfxA and near the boiling temperature of the ethanol. The result showed at 70°C, the specific activity of M2-N32G-S33P (1249 IU/mg) was also comparable to that of TfxA (1276 IU/mg) (Fig. 5B). Our experiment was a proof-of-concept that the N-terminus, particularly the key residues in Region 2 and 4, of TfxA, can be used, but not limited, to improve the thermostability of the xylanase SoxB.

Many previous studies have been suggested that it was the combined effect of many mutations and certain structural features that conferred stability on proteins in high temperature [37]. Here, seven mutations in the extreme N-terminus in combination conferred hyperthermostability on mesophilic xylanase SoxB. In the Region 2, all five mutations were involved in conferring the thermostability on the SoxB. The five mutations seemed to confer the thermostability on SoxB through both additive (the thermostabilizing effect of the individual mutations T11Y, N12H and Y16F) and cooperative (the H12-D13 electrostatic interaction and Y11-F16 hydrophobic interaction) effects [10]. However, in the Region 4, not all but two mutations, N32G and S33P, cooperated to strengthen the structural stability of SoxB, thus improving the thermostability of this xylanase. On the other hand, the M2-N32G-S33P did not exhibit significant difference with SoxB regarding the interaction between the two thermostabilizing regions. Thus, it was likely that the two thermostabilizing regions conferred hyperthermostability on SoxB by additive rather than cooperative effect. Taken together, five beneficial mutations in Region 2 and two beneficial mutations in Region 4 strengthened the structural stability on Region 2 and Region 4, respectively. Then, these two thermostabilizing regions in combination strengthen the overall structure of N-terminus and improved the thermostability of SoxB.

## Conclusions

This work provided a convincing example that mutations in the extreme N-terminus could confer thermostability (or even hyperthermostability) on a mesophilic xylanase. Moreover, this study further clarified the potential mechanism of thermostabilizing effect of the N-terminus of TfxA. It was demonstrated that not all divergent regions but two thermostabilizing regions were essential and played a dominant role in conferring the thermostability. In addition, a series of xylanases with a wide range of thermostabilities were obtained in this work and would provide flexible options for the deconstruction of lignocellulosic biomass.

## Supporting Information

Figure S1 **Sequence alignment of TfxA, SoxB and the mutant M2-N32G-S33P.** The divergent regions in N-terminus were highlighted by square frames and seven substitutions of M2-N32G-S33P were marked by stars.
(DOCX)

Table S1 **Sequences of oligonucleotides used in this work.**
(DOCX)
