# Supplementary material for: Seven N-terminal Residues of a Thermophilic Xylanase Are Sufficient to Confer Hyperthermostability on Its Mesophilic Counterpart
Source: PLoS One. 2014 Jan 30;9(1):e87632. doi: 10.1371/journal.pone.0087632 (PMC3907472; doi:10.1371/journal.pone.0087632)
Supplement: Figure S1 — Sequence alignment of TfxA, SoxB and the mutant M2-N32G-S33P. The divergent regions in N-terminus were highlighted by square frames and seven substitutions of M2-N32G-S33P were marked by stars. (DOCX) [file pone.0087632.s001.docx]

**R1 R2 R3 R4**
