# Supplementary material for: Seven N-terminal Residues of a Thermophilic Xylanase Are Sufficient to Confer Hyperthermostability on Its Mesophilic Counterpart
Source: PLoS One. 2014 Jan 30;9(1):e87632. doi: 10.1371/journal.pone.0087632 (PMC3907472; doi:10.1371/journal.pone.0087632)
Supplement: Table S1 — Sequences of oligonucleotides used in this work. (DOCX) [file pone.0087632.s002.docx]

**Supplementary Table**

Table S1. Sequences of oligonucleotides used in this work.

| Name | Sequence |
| --- | --- |
| pET-F | CGATCCCGCGAAATTAATACGACTC |
| pET-R | ATGCTAGTTATTGCTCAGCGGTGG |
| T30E-F | GGTTCGGTCTCGATGGAGCTGAACTCCGGCGGC |
| T30E-R | GCCGCCGGAGTTCAGCTCCATCGAGACCGAACC |
| N32G-F | GTCTCGATGACCCTGGGCTCCGGCGGCAACTAC |
| N32G-R | GTAGTTGCCGCCGGAGCCCAGGGTCATCGAGAC |
| S33P-F | TCGATGACCCTGAACCCTGGCGGCAACTACAGC |
| S33P-R | GCTGTAGTTGCCGCCAGGGTTCAGGGTCATCGA |
| T30E-N32G-F | GGTTCGGTCTCGATGGAGCTGGGCTCCGGCGGCAACTAC |
| T30E-N32G-R | GTAGTTGCCGCCGGAGCCCAGCTCCATCGAGACCGAACC |
| N32G-S33P-F | GTCTCGATGACCCTGGGCCCTGGCGGCAACTACAGC |
| N32G-S33P-R | GCTGTAGTTGCCGCCAGGGCCCAGGGTCATCGAGAC |
| T30E-S33P-F | GGTTCGGTCTCGATGGAGCTGAACCCTGGCGGCAACTACAGC |
| T30E-S33P-R | GCTGTAGTTGCCGCCAGGGTTCAGCTCCATCGAGACCGAACC |
| M2-M4-F | GGTTCGGTCTCGATGGAGCTGGGCCCTGGCGGCAACTACAGC |
| M2-M4-R | GCTGTAGTTGCCGCCAGGGCCCAGCTCCATCGAGACCGAACC |
| M2-N32G-S33P-F | GGTTCGGTCTCGATGACCCTGGGCCCTGGCGGCAACTACAGC |
| M2-N32G-S33P-R | GCTGTAGTTGCCGCCAGGGCCCAGGGTCATCGAGACCGAACC |
| TfxA-F | GAGATATACATATGGCCGTGACCTCCAACGAGACCGGGT |
| TfxA-R | CCGGAATTCTTAACCGCCGCTGGTGCCCAACGTC |
